# Integrated Treatment of PTSD and Substance Use Disorders: The Mediating Role of PTSD Improvement in the Reduction of Depression

**DOI:** 10.3390/jcm6010009

**Published:** 2017-01-13

**Authors:** Kristina J. Korte, Kaitlin E. Bountress, Rachel L. Tomko, Therese Killeen, Megan Moran-Santa Maria, Sudie E. Back

**Affiliations:** 1 Psychiatry Department, Massachusetts General Hospital, Harvard Medical School, Boston, MA 02114, USA; kkorte@mgh.harvard.edu; 2 Department of Psychiatry & Behavioral Sciences, Medical University of South Carolina, Charleston, SC 29401, USA; bountress@musc.edu (K.E.B.); tomko@musc.edu (R.L.T.); killeen@musc.edu (T.K.); moranm@musc.edu (M.M.-S.M.); 3 Ralph H. Johnson Veterans Affairs Medical Center, Charleston, SC 29401, USA

**Keywords:** posttraumatic stress disorder, PTSD, substance use disorders, alcohol, depression, integrated treatments, prolonged exposure, mediation, treatment

## Abstract

Posttraumatic stress disorder (PTSD) represents one of the most common mental health disorders, particularly among veterans, and is associated with significant distress and impairment. This highly debilitating disorder is further complicated by common comorbid psychiatric disorders, such as substance use disorders (SUD). Individuals with PTSD and co-occurring SUD also commonly present with secondary symptoms, such as elevated depression. Little is known, however, about how these secondary symptoms are related to treatment outcome. The aim of the present study, therefore, was to examine (1) the effects of treatment of comorbid PTSD/SUD on depressive symptoms; and (2) whether this effect was mediated by changes in PTSD severity or changes in SUD severity. Participants were 81 U.S. military veterans (90.1% male) with PTSD and SUD enrolled in a randomized controlled trial examining the efficacy of an integrated, exposure-based treatment (*Concurrent Treatment of PTSD and Substance Use Disorders Using Prolonged Exposure*; *n* = 54) versus relapse prevention (*n* = 27). Results revealed significantly lower depressive symptoms at post-treatment in the COPE group, as compared to the relapse prevention group. Examination of the mechanisms associated with change in depression revealed that reduction in PTSD severity, but not substance use severity, mediated the association between the treatment group and post-treatment depression. The findings underscore the importance of treating PTSD symptoms in order to help reduce co-occurring symptoms of depression in individuals with PTSD/SUD. Clinical implications and avenues for future research are discussed.

## 1. Introduction

Posttraumatic stress disorder (PTSD) represents one of the most common mental health disorders, with approximately 6%–8% of the general population [1,2] and 10%–30% of veterans [3,4,5] meeting lifetime diagnostic criteria. PTSD is associated with significant distress and impairment [6,7] and is further complicated by common comorbid psychiatric disorders, such as alcohol and drug use disorders [2].

The co-occurrence of PTSD and substance use disorders (SUD) is highly prevalent. A nationally representative sample of the United States population estimates that 46.4% of individuals with PTSD have a comorbid alcohol or drug use disorder [2]. PTSD/SUD comorbidity is associated with more severe PTSD symptoms [8,9], increased psychiatric comorbidity [10], distinct predictors of SUD relapse [11], and greater risk of relapse during or after SUD treatment [12,13,14]. PTSD/SUD comorbidity is also associated with increased dysthymia and depressive symptoms as compared to SUD-only samples [10,11] and, to a lesser extent, PTSD-only samples [10].

In clinical settings, patients presenting for treatment with PTSD/SUD comorbidity are commonly referred to SUD-only treatment first, and some are later referred to PTSD treatment [15,16]. However, prior research shows that integrated and concurrent treatments for PTSD/SUD can effectively reduce symptoms of PTSD while also significantly decreasing symptoms of SUD [12,17,18,19,20]. It may be that integrated treatments are more effective for complex symptom presentations, such as that observed in PTSD/SUD comorbidity, than treatments designed to treat one disorder exclusively. Importantly, reductions in PTSD symptoms following treatment of PTSD/SUD are associated with corresponding reductions in SUD symptoms [12,18,19]. In contrast, reductions in SUD symptoms have not been associated with corresponding reductions in PTSD symptoms [12,18]. Further, recent evidence has emerged showing that the change in PTSD symptoms predicts change in SUD symptoms during integrated treatment; however, a reduction in SUD symptoms only minimally predicts change in PTSD symptoms during treatment [18,21,22]. In a recent meta-analysis, Roberts and colleagues (2015) found that trauma-focused treatment administered alongside a SUD treatment was more efficacious than treatment as usual; however, they noted that the quality of the existing literature was low. Thus, studies focusing on the treatment of PTSD/SUD are of the utmost importance to improve the existing literature base.

The role of aversive emotional states in the etiology, maintenance of, and relapse to alcohol and drugs is well established [23,24,25]. Consistent with negative reinforcement models of alcohol and drug use, individuals with PTSD endorse use of alcohol and other substances to alleviate PTSD-related symptoms [26,27] and perceive a relationship between their PTSD symptoms and substance use [15,21]. If substance use reduces stress or allows the individual to avoid unwanted PTSD-related thoughts or feelings, the substance use is negatively reinforced and more likely to continue in the future. Thus, to the extent that individuals with PTSD use substances to “self-medicate” PTSD-related symptoms, PTSD may be more likely to maintain SUD symptoms, rather than vice versa.

Individuals with PTSD/SUD comorbidity may also use substances to alleviate secondary symptoms, such as depression. Although individuals with PTSD/SUD comorbidity commonly present with elevated depressive symptoms [10,11], little is known about how this impacts treatment outcomes, or which treatment approaches are optimal in the reduction of secondary, but highly related symptoms such as depression. Given the elevated rates of depression among individuals with PTSD/SUD comorbidity, it is important to consider how reductions in PTSD and SUD symptoms impact symptoms of depression. A recent review [28] suggests that exposure-based treatments for PTSD may be effective in reducing co-occurring depressive symptoms. However, it is not known whether integrated PTSD/SUD treatments would have similar efficacy for depressive symptoms. Both PTSD and SUD may serve to exacerbate or maintain symptoms of depression [25,28]. In particular, it is possible that an integrated intervention targeting PTSD/SUD concurrently may have a greater impact on the reduction of depression than SUD treatment alone.

The aim of the present study was to examine the effects of integrated treatment for PTSD/SUD, as compared to SUD-only treatment, on symptoms of depression among individuals with current PTSD and SUD. Further, we sought to evaluate the potential underlying mechanisms associated with the reduction in depression. Given the accumulating literature demonstrating that a reduction in PTSD symptoms directly impacts a reduction in SUD symptoms, and not vice versa [12,18,22], we examined whether improvement in depressive symptoms was mediated by a reduction in PTSD or SUD symptoms. These questions were evaluated using data from a randomized controlled trial comparing the effects of an integrated, exposure-based treatment for PTSD and SUD (*Concurrent Treatment of PTSD and Substance Use Disorders Using Prolonged Exposure*; COPE) [29] in comparison to relapse prevention (RP) [22]. It was hypothesized that the COPE group would demonstrate a greater reduction in depressive symptoms than the RP group. In addition, we hypothesized that the change in PTSD and SUD symptoms would mediate the change in depressive symptoms.

## 2. Method

### 2.1. Participants

Participants were 81 veterans with the current *Diagnostic and Statistical Manual of Mental Disorders-IV* (DSM-IV) [30] diagnoses of PTSD and SUD who participated in a randomized controlled trial. Veterans were recruited from flyers placed in local VA and community treatment clinics, newspaper and internet advertisements (e.g., Craigslist), and clinician referrals. Eligibility criteria included: (1) being a veteran; (2) between the ages of 18–65 years old; (3) meeting DSM-IV criteria for current PTSD and a having a total score ≥ 50 on the Clinician Administered PTSD Scale for the DSM-IV (CAPS) (4) meeting DSM-IV criteria for a current SUD (i.e., alcohol or substance abuse or dependence disorder) [30] and (5) use of alcohol or other substances within the 90 days prior to study enrollment. Participants were also required to be stabilized on any psychotropic medications for at least four weeks before beginning the study. Individuals were excluded for current suicidal or homicidal ideation and intent, a history of current psychotic or bipolar affective disorders, and eating disorders as these would likely require a higher level of care. Individuals already receiving psychosocial treatment for PTSD or SUD were also excluded from participation.

### 2.2. Procedure

Potential participants were screened for eligibility either by phone or in person. After reading and signing an Internal Review Board-approved informed consent, eligible participants were randomized (2:1) to receive 12 individual therapy sessions of either COPE (*n* = 54) or RP (*n* = 27). We utilized a 2:1 randomization procedure to ensure that we would have sufficient power to examine the efficacy of COPE since it is a new treatment approach. Participants were stratified across the treatment conditions by baseline severity of PTSD and substance use. Participants received $20 compensation for participation in each session during the course of treatment.

### 2.3. Treatments

COPE is a manualized, integrated cognitive behavioral therapy for comorbid PTSD and SUD that consists of 12 weekly, individual, 90 min sessions [29]. Sessions 1–3 focus on goal-setting, psychoeducation related to PTSD and SUD, methods for coping with cravings and introduction to prolonged exposure. In vivo exposure is introduced in session 3 and continues through session 12. Imaginal exposure begins in session 4 and continues until session 11 (eight total imaginal exposure sessions lasting 30–45 min each). Skills for managing thoughts about using and triggers, as well as drink/drug refusal skills are integrated into the treatment. Each session is recorded and patients are asked to listen to the full session once a week and the imaginal exposure recordings daily.

Relapse prevention (RP) is a manualized cognitive behavioral therapy used for the treatment of SUD that consisted of 12 weekly, individual, 90 min sessions [31]. RP focuses on teaching patients a variety of recovery-related skills. In RP, patients learn to identify triggers associated with their use, and learn ways to manage cravings, high-risk situations, and anger. Treatment topics related to SUD were manualized to be similar across the two treatment groups. RP sessions were also videotaped and screened for fidelity, as well as verifying that clinicians did not include components of treatment for PTSD.

The therapy interventions were delivered by doctoral-level psychologists (*n* = 6) or masters-level clinicians (*n* = 1). Each clinician completed a two- to three-day training workshop that included a review of the treatments, instructions on the treatment techniques, practice exercises, and role plays. Throughout the duration of the study, clinicians received weekly supervision (T. Killeen or S.E. Back). All therapy sessions were videotaped and rated using adherence and competency measures adapted from previous studies [17]. The COPE and RP treatments were administered by the same clinicians to reduce potential therapist related confounds.

### 2.4. Measures

PTSD symptoms. The Clinician Administered PTSD Scale for the DSM-IV (CAPS) [32] was used to assess current PTSD diagnoses and symptomatology. A total score of 50 or higher at baseline was required for study inclusion. The CAPS assessed for frequency and intensity of PTSD symptom clusters including avoidance, re-experiencing, and hyperarousal symptoms. The CAPS also measured impairment in functioning and symptoms commonly associated with PTSD (e.g., guilt). The CAPS demonstrated good reliability in the present study (Cronbach’s alpha = 0.85).

The PTSD Checklist-Military (PCL-M) [33], a 17-item self-report questionnaire, was used to assess PTSD symptom severity. Veterans rated how much they had been bothered by symptoms on a Likert scale ranging from 1 (*Not at all*) to 5 (*Extremely*). The PCL excellent internal consistency, test-retest reliability, and good convergent validity with related PTSD measures [33]. The PCL-M was administered at baseline to assess for PTSD symptoms severity prior to starting treatment. The PCL was also administered weekly during the study to assess for changes in symptoms at mid-treatment and post-treatment. The PCL demonstrated good reliability in the present study (Cronbach’s alpha = 0.87).

Substance Use. The Mini International Neuropsychiatric Interview (MINI) [34] is a semi-structured clinician administered interview that was used to assess for current alcohol or substance use disorder. The MINI was also used to assess for potential exclusionary mental health diagnoses. The MINI was administered at pre and post-treatment. The MINI has been shown to have sound psychometric properties [34].

The Timeline Follow-Back (TLFB) [35] was used to assess the frequency (percent days using substances/drinking alcohol) and quantity (amount of substance use and standard drink units consumed per day) of alcohol and substance use (e.g., cocaine, marijuana, opioids, prescription drugs, and sedatives). The TLFB method obtains retrospective self-report of alcohol and substance use by using a calendar and other memory prompts to stimulate recall. The TLFB assessed for alcohol and substance use 60 days prior to initiating treatment and weekly throughout the course of treatment. The TLFB has sound psychometric properties with excellent test-retest reliability and good confirmatory and discriminatory validity (Carey, 1997). A ratio of the number of days using to the number of days assessed was computed in order to capture the proportion of using days in the past 60 days.

Depressive Symptoms. The BDI-II is a self-report questionnaire consisting of 21 items assessing symptoms of depression [36]. The BDI-II has sound psychometric properties including excellent reliability and validity [37,38]. The BDI was also administered weekly during treatment to assess for change in depressive symptoms. The BDI demonstrated excellent reliability in the present study (Cronbach’s alpha = 0.92).

### 2.5. Data Analytic Plan

To test the hypothesized effects, a path model was estimated using Mplus Version 7 using the Model Indirect command [39]. All continuous covariates/predictors were centered prior to conducting analyses. Missing data on endogenous variables was estimated as a function of the observed exogenous variables under the missingness at random assumption.

Age, gender, race, baseline PTSD symptoms, baseline alcohol and substance use (as a composite variable) and baseline depression symptoms were used as covariates in predicting the intermediate outcomes (i.e., mediators) of PTSD symptoms and alcohol use and substance use at session 6 (mid-treatment). These covariates were selected to control for any between group differences in demographics and the primary outcome variables at baseline. The main effect of treatment was entered as a hypothesized predictor. PTSD symptoms and alcohol use and substance use at session 6, as well as treatment condition, were then used to predict depression symptoms at the session 12 (end of treatment). Interactions between group and gender, age and race were tested to ensure that the intervention had equivalent effects across individuals with varying demographic characteristics. Interactions that were not significant (*p* < 0.05) were trimmed from the model. The indirect effects of treatment on depression symptoms at session 12 through PTSD symptoms and SUD symptoms at the session 6 were also examined.

## 3. Results

### 3.1. Baseline Demographics and Clinical Characteristics

As shown in Table 1, participants were predominantly male (90.1%) with a mean age of 40.4 years (*SD* = 10.7). The majority were Caucasian (60.5%), followed by 37.0% African American, 3.7% Hispanic, and 2.5% other. Most participants (81.0%) reported that their index trauma was military-related (e.g., combat exposure, accident during the military, military sexual trauma). Participants had an average of 13.9 years of education (*SD* = 2.0) and primarily served in Operation Enduring Freedom, Operation Iraqi Freedom, or Operation New Dawn (63.7%).

Diagnoses. All eligible participants met DSM-IV criteria for PTSD and a co-occurring SUD. The majority of participants (63.0%) met criteria for an alcohol use disorder (alcohol dependence: *n* = 47, alcohol abuse: *n* = 4), 27.2% met criteria for an alcohol use disorder and at least one drug use disorder, and 9.9% met criteria for a drug use disorder but no alcohol use disorder.

### 3.2. Final Model Results

Goodness of fit was determined by comparing results from the model with standards for acceptability [40]. Model results are presented in Table 2. This model showed good fit to the data: root mean square error of approximation (RMSEA) = 0.081, standardized root mean square residual (SRMR) = 0.33 and comparative fit index CFI = 0.954. In examining PTSD symptoms at session 6, the covariates of age, race, and baseline PTSD symptoms conferred risk. Specifically, older adults reported more severe PTSD symptoms at session 6 (*p* < 0.01), as did Caucasians (*p* < 0.05), and individuals who reported more severe PTSD symptoms at baseline (*p* < 0.01). Additionally, the treatment group was significantly (*p* < 0.05) associated with PTSD symptoms at session 6. Specifically, individuals who received RP reported more severe PTSD symptoms, compared to individuals who received COPE (see Table 1).

In predicting substance use at session 6, only substance use at baseline was associated with this outcome (*p* < 0.01). Specifically, individuals who reported more severe substance use at baseline also reported higher substance use at session 6. No other covariates or the treatment condition were associated with substance use at session 6.

In predicting depressive symptoms at session 12, only PTSD symptoms were predictive. Specifically, more severe PTSD symptoms at session 6 were associated with more severe depressive symptoms at session 12. Neither substance use at session 6 nor treatment condition predicted later depressive symptoms.

### 3.3. Mediators of Change in Depression

**Figure 1:**
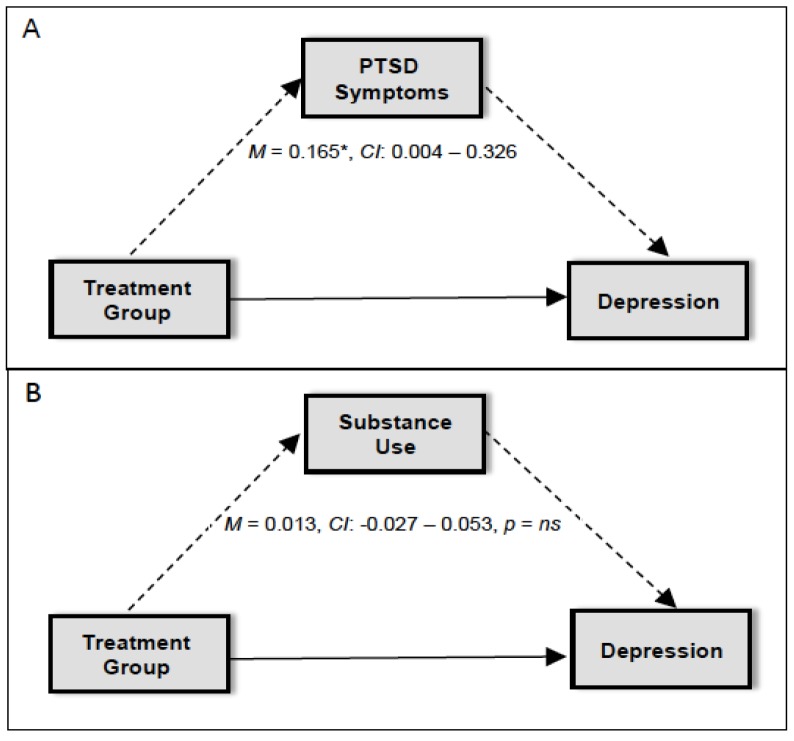
Diagram for the mediation model for (**A**) PTSD symptoms and (**B**) substance use as a mediator in the association between treatment group and depression. * *p* < 0.05.

We examined the indirect effect of the treatment group on depressive symptoms at session 12 via (1) change in PTSD symptoms at session 6; and (2) change in substance use at session 6. Results indicated that PTSD symptoms were a significant mediator of the effect of the treatment group on depressive symptoms (standardized estimate: 0.165, 95% CI: 0.004–0.326, *p* < 0.05). This finding indicates that (1) individuals who received RP endorsed more severe PTSD symptoms at session 6 compared to individuals who received COPE; and (2) individuals with more severe PTSD symptoms at session 6 reported more severe depressive symptoms at session 12 (see Figure 1). However, substance use was not a significant mediator of the effect of the treatment group on depressive symptoms (standardized estimate: 0.013, 95% CI: −0.027–0.053, *NS*; see Figure 1). Individuals in both treatments evidenced similar levels of reduction in substance use, and reduction in substance use during treatment did not influence later depressive symptoms.

## 4. Discussion

The present study examined the effect of treatment for PTSD and SUD on secondary treatment outcomes, as well as the underlying mechanisms of change. In particular, we examined (1) the effect of treatment on change in depressive symptoms; and (2) whether this effect was mediated by changes in PTSD severity or changes in SUD severity. Overall, the results were consistent with a priori hypotheses and demonstrated that the integrated treatment, as compared to the SUD-only treatment, was more effective at reducing symptoms of depression in patients with PTSD/SUD. This is particularly important, as depressive symptoms tend to be elevated in this complex clinical presentation and can serve as a trigger for substance use. This finding is consistent with the existing literature demonstrating that PTSD treatments, such as prolonged exposure (PE) [41], are effective at impacting secondary outcomes of treatment such as depressive symptoms [41,42,43]. Moreover, this is the first study to our knowledge demonstrating this effect among individuals with PTSD and co-occurring SUD receiving an integrated, exposure-based intervention (i.e., COPE), and the first to demonstrate the specificity of the effect to the integrated treatment, as compared to the SUD-only treatment.

Examination of the potential underlying mechanisms driving the reduction in depression revealed interesting and clinically relevant findings. Specifically, we examined whether the change in depressive symptoms at post-treatment was mediated by a reduction in PTSD symptoms or a reduction in substance use severity. Consistent with the principles of establishing mediation [44], the present study demonstrated temporal precedence of the change in PTSD symptoms at mid-treatment mediating the change in depressive symptoms at post-treatment. In contrast, the mid-treatment change in substance use symptoms did not mediate the change in post-treatment depressive symptoms, thereby further bolstering the significance of these findings and the benefits of integrated PTSD/SUD treatment on depression.

Although it can be argued that the effect of PTSD symptoms in mediating the change in depressive symptoms is likely to be somewhat influenced by the overlapping diagnostic symptoms associated with PTSD and depression (i.e., anhedonia, trouble concentrating, difficulty sleeping), the effect observed is also likely influenced by other overlapping, more generalized features of these disorders, such as the shared underlying temperamental and genetic vulnerabilities (i.e., neuroticism) [45]. However, despite the tendency for individuals with substance use disorders to also have symptoms that overlap with depression (e.g., trouble concentrating, sleep problems), the same pattern was not observed with the substance use as a mediator, thereby suggesting that the change in substance use may be more driven by indirect mechanisms, such as changes in PTSD symptoms that influence PTSD/SUD comorbidity [46].

The findings of the study add to the accumulating evidence for the benefits of treating PTSD and SUD concurrently, as the impact of integrated treatment may expand beyond the reduction of PTSD and SUD symptoms by also reducing secondary symptoms such as depression. This is important to highlight given that there is no consensus regarding whether sequential or integrated treatments are optimal for PTSD/SUD. The study provides further support for the benefits of using integrated treatment approaches as it appears that a sequential approach to treatment may unnecessarily extend the time of suffering from PTSD *and* symptoms of depression, whereas integrated treatments can impact the symptoms of PTSD, SUD and depression concurrently.

### 4.1. Limitations

The present study should be considered in light of several limitations. Participants in the study were comprised primarily of male veterans with combat-related PTSD and a co-occurring alcohol use disorder, thereby potentially limiting the generalizability of the findings. It is notable that the COPE group had a larger percentage of White participants than the RP group. To address this potential limitation, we controlled for race in the analyses. Further, the use of self-report data and retrospective reporting may have influenced the results. Future research should include the use of biological approaches (e.g., biomarkers) with self-report questionnaires to enhance the assessment of alcohol and substance use during the course of treatment. Future research would also benefit from replicating these findings in more diverse samples, such as women and civilian-related traumas. It could also be argued that use of the same therapists across the COPE and RP groups could have influenced the results. To minimize this potential confounding factor, we assessed for treatment fidelity across the COPE and RP sessions to ensure the treatments were sound across the groups. That being said, this limitation is somewhat ameliorated in the current study in that a substantial portion of the participants also experienced civilian-related traumas in addition to their military-related index trauma. Despite this, veteran populations with military trauma are a distinct group, which underscores the importance of expanding this work to other groups to enhance the generalizability of the findings.

### 4.2. Future Directions

The results from the present study provide several avenues for future investigation. First, it important to examine the whether the change in the primary outcomes, PTSD and SUD, observed at post-treatment is maintained during follow-up. It is also crucial to assess the maintenance of the reduction in depressive symptoms over time. Some evidence indicates that the reduction in depression symptoms is maintained at follow-up in trials of PE for PTSD [42]. As such, it will be important to see if the change in depressive symptoms is also maintained when using PE as an integrated intervention for individuals with co-occurring SUD. Future research would also benefit from examining the impact of integrated treatments on other secondary outcome measures, such as anxiety and suicidality. This line of work could provide further insight into whether changes in other related but distinct constructs are influenced by the change in PTSD symptoms or substance use, thereby ultimately providing a better understanding of the underlying mechanisms associated with improvement in secondary outcomes in PTSD/SUD. Finally, given the tendency for providers in community settings to utilize sequential approaches to treating PTSD/SUD, it is also crucial to compare the cost-effectiveness of integrated treatments, such as COPE, to sequential treatment approaches. It stands to reason that treating multiple disorders concurrently, such as PTSD/SUD, and the secondary symptoms such as depression, may be more cost-effective than treating disorder-specific symptoms in a sequential treatment design.

## Figures and Tables

**Table 1:** Demographics and primary outcome measures among participants in the COPE and RP groups.

Variable	COPE	RP	Total
	(*n* = 54)	(*n* = 27)	(*n* = 81)
	M (*SD*) or %	M (*SD*) or %	M (*SD*) or %
Age (Years)	39.7 (10.9)	41.9 (10.3)	40.4 (10.7)
Gender (Male)	92.6%	85.20%	90.1%
Race (Caucasian)	68.50%	44.40%	60.5%
Race (African American)	29.60%	51.90%	37.0%
BDI			
Baseline BDI	29.2 (12.3)	29.6 (9.7)	29.3 (11.5)
Session 6 BDI	19.5 (11.7)	26.2 (13.7)	21.3 (12.5)
Session 12 BDI	13.0 (11.0)	19.4 (12.3)	15.02 (11.7)
PCL			
Baseline PCL	62.2 (11.1)	64.3 (8.9)	62.9 (10.4)
Session 6 PCL	45.5 (15.6)	58.0 (18.5)	48.9 (17.2)
TLFB			
Baseline TLFB	0.47 (0.36)	0.50 (0.34)	0.48 (0.35)
Session 6 TLFB	0.21 (0.26)	0.29 (0.30)	0.23 (0.27)

Note: COPE, Concurrent Treatment of PTSD and Substance Use Disorders Using Prolonged Exposure; RP, Relapse Prevention; BDI, Beck Depression Inventory Total Score; PCL, PTSD Checklist Total Score; TLFB, Timeline Follow Back percent days using; M, mean; SD, Standard Deviation.

**Table 2:** Standardized model results.

Predictor	Session 6 PTSD Symptoms		Session 6 Substance Use		Session 12 Depressive Symptoms	
	B	SE	B	SE	B	SE
Age	0.370 **	0.103	−0.220	0.114		
Gender	−0.046	0.109	0.054	0.118		
Race	−0.208 *	0.089	−0.027	0.097		
Treatment Group	0.236 *	0.112	0.136	0.122	−0.072	0.123
Baseline PTSD Symptoms	0.405 **	0.126	-0.052	0.141		
Baseline Substance Use	−0.056	0.109	0.477 **	0.104		
Session 1 Depressive Symptoms	0.118	0.120	0.019	0.131		
Session 6 PTSD Symptoms	--	--	--	--	0.700 ***	0.091
Session 6 Substance Use	--	--			0.096	0.122

Notes: *N* = 81; *** *p* < 0.001, ** *p* < 0.01, * *p* < 0.05; Gender: 0 = males and 1 = females; Race: 0 = Caucasian, 1 = African American or Other; Treatment group: 0 = COPE, 1 = RP.
